# *In silico *modeling of the pore region of a KCNQ4 missense mutant from a patient with hearing loss

**DOI:** 10.1186/1756-0500-5-145

**Published:** 2012-03-15

**Authors:** Kazunori Namba, Hideki Mutai, Hiroki Kaneko, Sho Hashimoto, Tatsuo Matsunaga

**Affiliations:** 1 Laboratory of Auditory Disorders, National Institute of Sensory Organs, National Tokyo Medical Center, Tokyo, Japan; 2 Department of Integrated Science in Physics and Biology, College of Humanities and Science, Nihon University, Tokyo, Japan; 3 Department of Otolaryngology, National Sendai Medical Center, Miyagi, Japan

**Keywords:** Channel, Deafness, Electrostatic, Hearing loss, Molecular Modeling, Mutation, Potassium, Structure

## Abstract

**Background:**

Mutation of the voltage-gated potassium channel KCNQ4 causes DFNA2-type nonsyndromic autosomal dominant sensorineural hearing loss. KCNQ4 is expressed predominantly in the auditory sensory outer hair cells, which are critical for sound amplification.

**Results:**

We sequenced *KCNQ4 *from Japanese patients with sensorineural hearing loss, and identified a novel missense mutation encoding a Tyr270His located at the N-terminus of the highly conserved pore helix sequence. As this patient was not accessible to us and information about them was limited, we used molecular modeling to investigate whether this novel mutation is hypothetically pathogenic. A careful examination of an *in silico *structural model of the KCNQ4 pore region revealed that the Tyr270His mutation caused an alteration in the electrostatic surface potential of the pore helix.

**Conclusion:**

We propose two possible means by which the Tyr270His mutation causes hearing loss: a positively charged His270 side chain might enhance the helix dipole moment of the pore helix, thereby destabilizing the helix and/or the pore region, or it might disturb transport of K^+ ^through the channel by electrostatic repulsion.

## Background

Deafness is a frequently inherited sensory disorder. For every 1000 newborns, more than 1% have bilateral sensorineural hearing loss (SNHL), and 50-70% of the cases are monogenic disorders [1,2]. Hereditary hearing loss is classified as syndromic and nonsyndromic [3]. The genetic causes of nonsyndromic hearing loss are autosomal dominant, autosomal recessive, X-chromosome linked, and mitochondrial in nature. To date, 25, 40, 3, and 6 genes have been identified that are responsible for autosomal dominant-, autosomal recessive-, X-chromosome linked-, and mitochondrial hearing loss, respectively http://hereditaryhearingloss.org/. In particular, autosomal dominant nonsyndromic sensorineural deafness type 2 (DFNA2) hearing loss affects the ability of children to hear high frequencies, and results in hearing loss at all frequencies later in life [4]. *KCNQ4 *is the causative gene [5] and encodes the membrane protein KQT-like, subfamily member 4, which contains 695 amino acid residues in its longest isoform.

KCNQ4 is a member of the five muscarinic receptor-regulated and voltage-gated potassium (M-type K^+ ^channel) channels present in ear and epithelia [6]. In the inner ear, KCNQ4 subunits assemble to form homomeric channels that help with the recycling of potassium [5]. Because patients with *KCNQ4 *mutations show progressive hearing loss, development of drugs that improve KCNQ4 function should attenuate hearing loss.

While conducting an ongoing genetic study of hereditary hearing loss, we identified a mutated *KCNQ4 *from a Japanese patient in which the encoded Tyr270 residue was replaced with His. Structural analysis of mutation**s **using molecular modeling is a practical technology to investigate their pathogenicity in patients who are not readily available for analysis. To determine how the mutated protein (denoted Tyr270His) induced hearing loss, we built models of the KCNQ4 and Tyr270His pore regions. By examining models that included electrostatic potentials, we were able to propose two molecular mechanisms that could have caused the patient's hearing loss.

## Results and discussion

**Figure 1:**
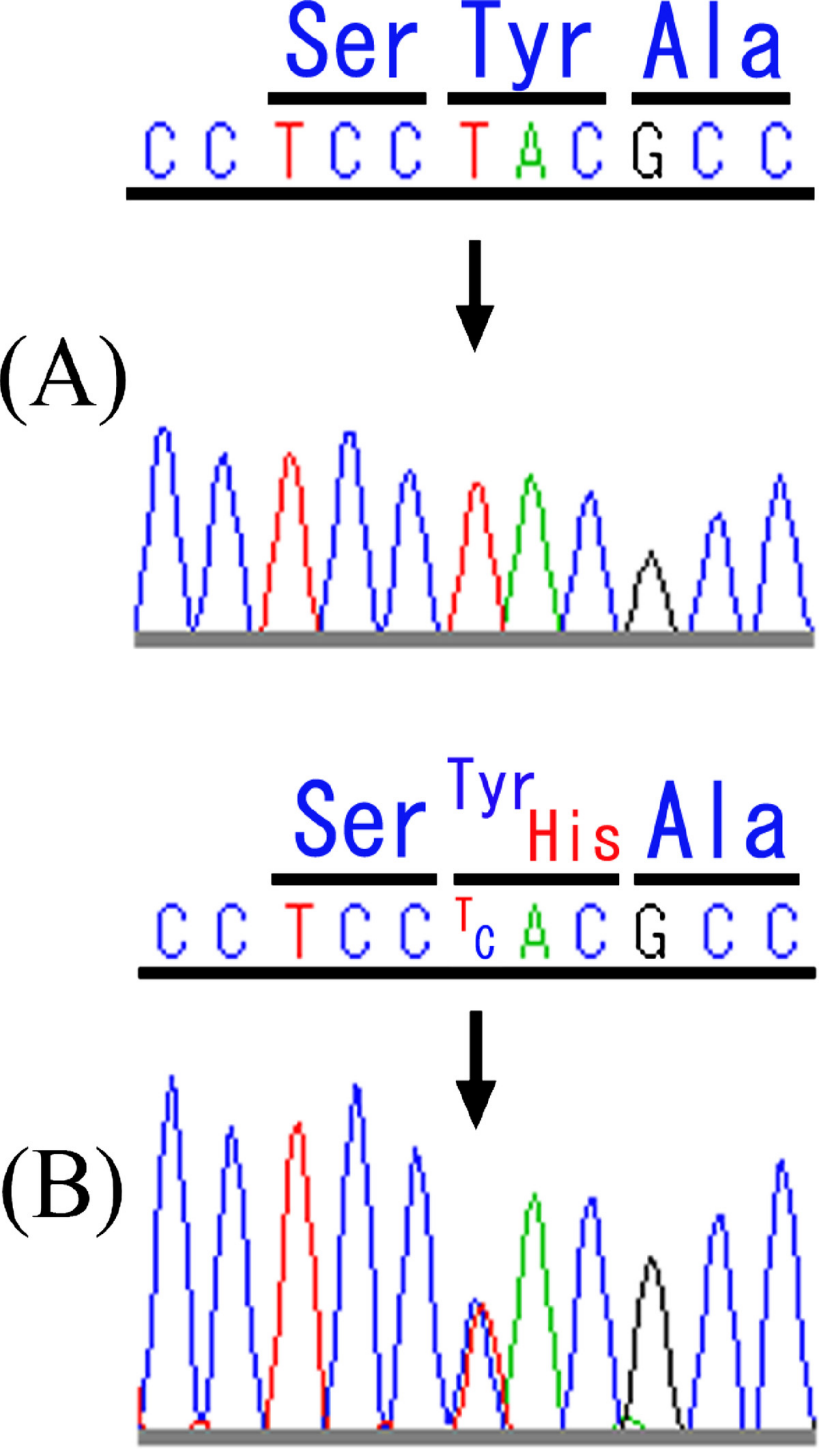
**Partial DNA-sequencing chromatograms of *KCNQ4 *exon 5 from (A) a subject with normal hearing and (B) the patient with Tyr270His**. The position of the heterozygotic T → C mutation at nucleotide position 808 is indicated by the arrow. This mutation was not found in 96 healthy control subjects with normal hearing. No additional mutations were identified in the *KCNQ4 *coding region of this mutated gene.

Prior to this report, 14 missense mutations, a splice-site mutation, and 2 small deletion mutations in *KCNQ4 *were reported to be associated with hearing loss [7-9]. Because 11 of the missense mutations were found in the pore region, it was considered a pathogenic "hot spot". We therefore sequenced *KCNQ4 *exons 5, 6, and 7 (which encode the pore region) of Japanese patients with bilateral nonsyndromic hearing loss. A new heterozygotically presented *KCNQ4 *missense mutation was found at position 808, in which the wild-type thymine (T) was replaced by cytosine (C). This results in a Tyr270His mutation in the protein (Figure 1).

**Figure 2:**
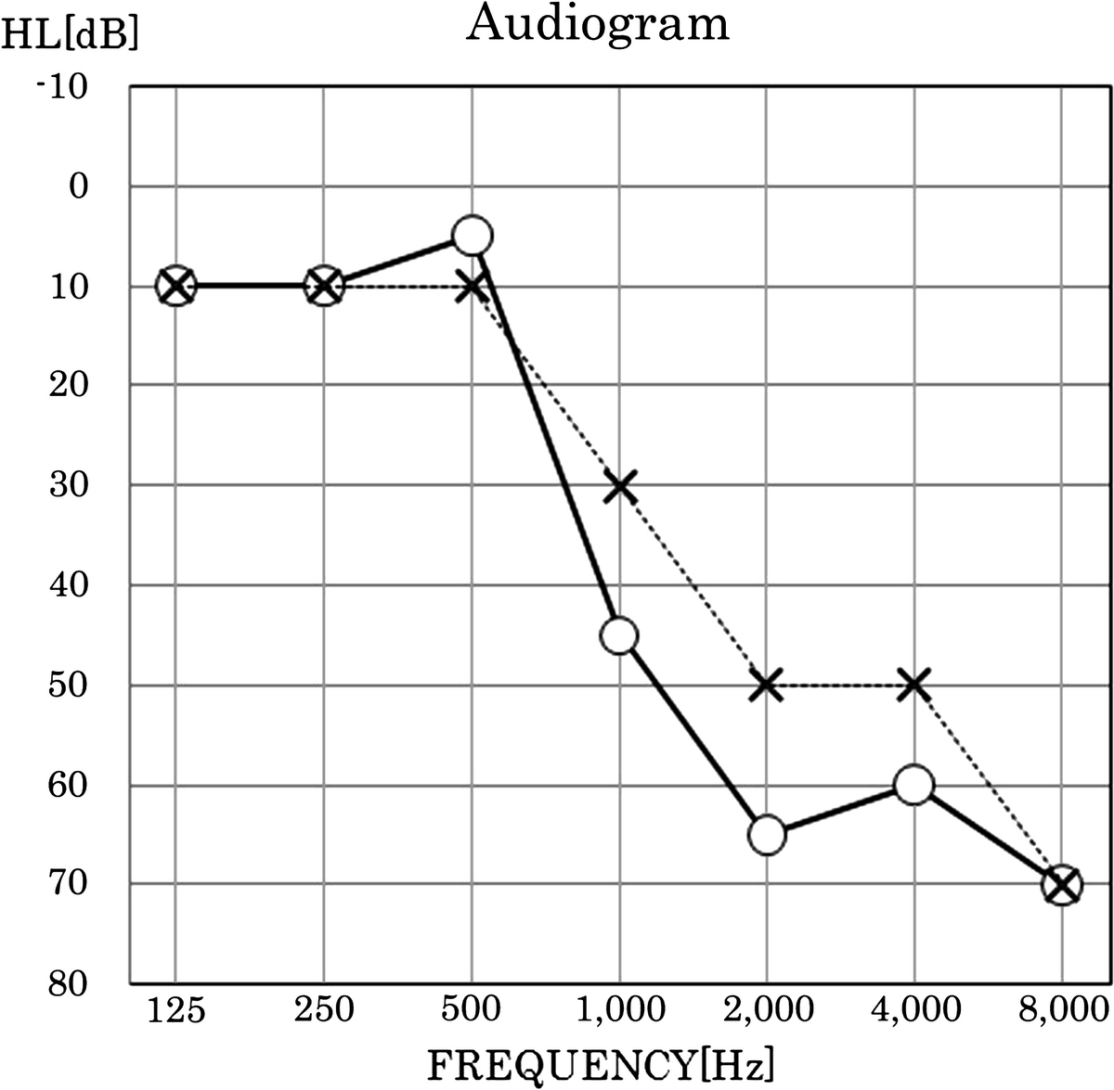
**An audiogram from the patient with Tyr270His**. Open circles connected by the solid line are the hearing levels for the right ear. Crosses connected by the dotted line are the hearing levels for the left ear.

The patient who carried the Tyr270His mutation was a 28-year-old female with congenital, progressive hearing loss. An audiometric examination found mild hearing loss in her left ear and moderate hearing loss in her right ear, with steeply sloping audiometric configurations in both ears (Figure 2). The patient had no inner ear malformations, according to computational tomography. None of her family members had hearing loss, and no environmental factors that might have caused the hearing loss were reported (*e.g*., exposure to noise or ototoxic drugs.) DNA samples were not obtained from her family.

**Figure 3:**
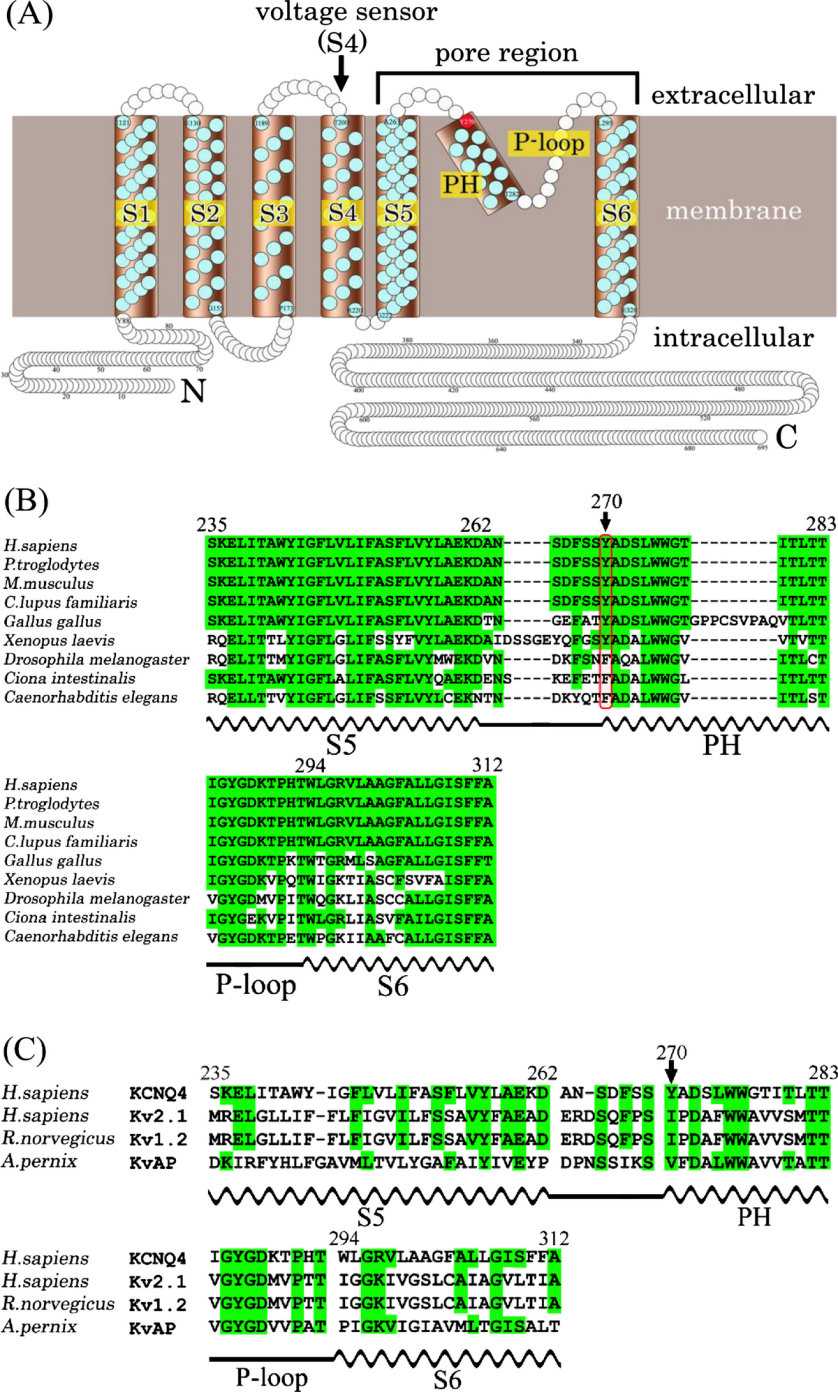
**Schematic topology and partial protein sequence of KCNQ4**. (A) Schematic topology represents six transmembrane helices (S1–S6) and the K^+^-selective channel pore region (S5, PH, P-loop, and S6) of KCNQ4. (B) Sequences of the orthologous KCNQ4 pore region are aligned. Positions highlighted in green indicate the amino acid is the same as in human KCNQ4. The position of Tyr270 is indicated by an arrow. The positions of S5, PH, S6 (wavy lines) and the P-loop (straight line) are shown below the sequences. (C) Alignment of the KCNQ4 pore-region sequence with those of Kv channels deposited in PDB. Positions highlighted in green indicate the amino acid is the same as in human KCNQ4. The sequence of human KCNQ4 is indicated at the top of both alignments.

Similar to other voltage-gated K^+ ^(Kv) channels, KCNQ4 probably contains six transmembrane α-helices, (S1-S6), a pore helix (PH), a pore-loop (P-loop), a short N-terminal region, and a long C-terminal region (Figure 3A). The pore regions of Kv channels are formed by the contiguous regions S5, PH, P-loop, and S6. Functional and structural characterizations of Kv channels have demonstrated that the PH and P-loop are responsible for selective K^+ ^transport, whereas S4, acting as a voltage sensor, regulates the open/closed state of the channel [10,11]. A modeling study of wild-type and mutant KCNQ3 channels suggested a critical stabilizing interaction between the PH and the S4-selectivity filter that is responsible for rearrangements of the P-loop architecture induced by the presence of a hydroxyl-containing residue at the 315 position, "unlocking" the channels into a conductive conformation [12]. Tyr270 is predicted to be the N-terminal residue of the PH. Available mammalian KCNQ4 pore-region sequences are identical, and those of non-mammals (*e.g*., birds, fruit flies, tunicates, and nematodes; Figure 3B) are very similar to mammalian sequences. In addition, the PH and P-loop sequences of non-KCNQ4 Kv channels (*e.g*., Kv2.1 from *Homo sapiens *is identical in 14/24 amino acid residues, Kv1.2 from *Rattus norvegicus *in 11/24, and KvAP, a prokaryotic Kv channel from *Aeropyrum pernix *in 13/24) are somewhat conserved, suggesting that the pore residues, including Tyr270, are necessary for the proper functioning of the channel (Figure 3C).

Given the patient's clinical data and that the Tyr270His mutation is within the highly conserved pore region, we built structural models of wild-type KCNQ4 and the Tyr270His pore regions in an attempt to understand how the mutation affected K^+ ^transport and, consequently, the patient's hearing. To identify a template crystal structure, we searched the Protein Data Bank using Gapped BLAST [13] and PDBsum [14], to obtain a three-dimensional structure of a KCNQ4 homolog. Among the channels with available three-dimensional structures, the Kv1.2 sequence was most similar to that of KCNQ4 (27.5% identity for the transmembrane sequence Ser32 to Thr417). Consequently, structural models for the transmembrane regions (Tyr80 to Gln329) of wild-type KCNQ4 and Tyr270His were built based on the template given by the crystal structure of Kv1.2 using SWISS-MODEL http://swissmodel.expasy.org/[15-17]. The reliabilities of the models were evaluated using Verify_3D http://nihserver.mbi.ucla.edu/Verify_3D/[18,19] , and were found to be trustworthy (see Additional file 1: Figure S1).

**Figure 4:**
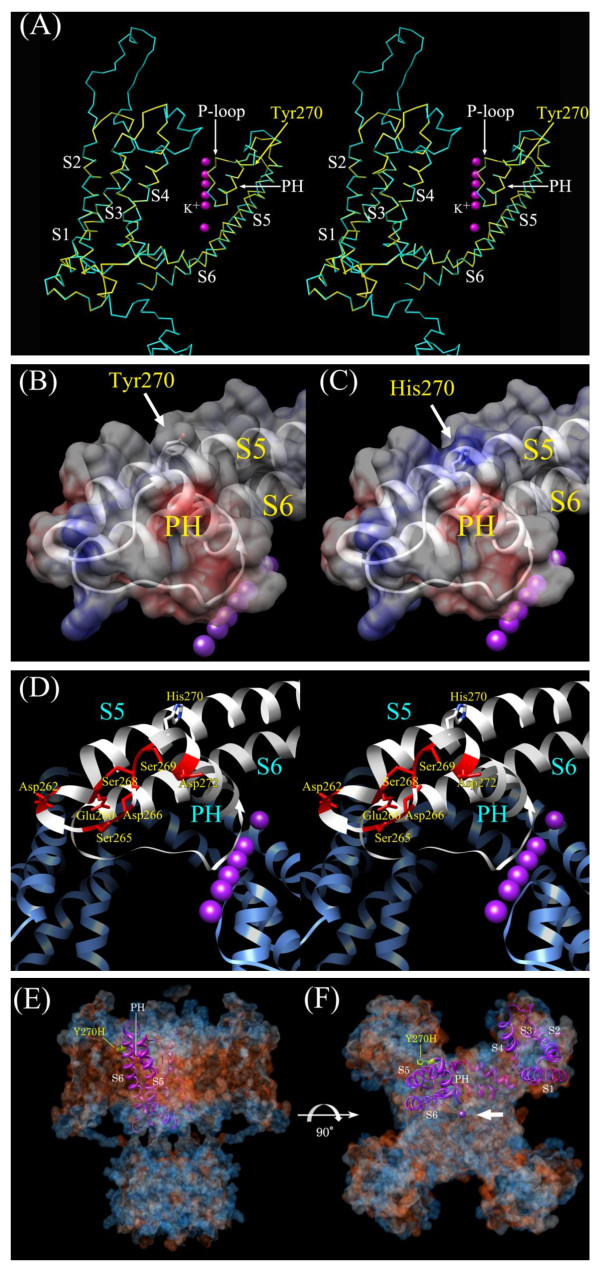
**Structural models of KCNQ4 and Tyr270His**. (A) Stereoview of the KCNQ4 α-carbon frame model (yellow) superimposed onto that of Kv1.2 (cyan). The position of Tyr270 is indicated with a yellow arrow. The α-helices, the PH, and the P-loop are also identified. (B) Part of the wild-type KCNQ4 model, and (C) the Tyr270His model overlaid with their corresponding electrostatic surface potentials. The side chains of Tyr270 (B) and His270 (C) are indicated by arrows. Negatively and positively charged residues are depicted respectively in red and blue in the electrostatic potential surface representations. (D) Stereoview of a portion of the ribbon model of the Tyr270His pore region. Residues that are negatively charged or that can form a hydrogen bond and surround His270 are identified and shown in red. (E, F) The Tyr270His ribbon model was superimposed onto (E) a horizontal view of the plasma membrane and (F) an extracellular view with the four rotational axes of the Kv1.2 crystal structure shown as hydrophobicity surface. Hydrophobic and hydrophilic residues are depicted respectively in red and blue in the hydrophobicity surface representations. His270 is shown in yellow. (A-F) K^+ ^in the central pore is shown as a purple sphere and indicated by an arrow in (F).

The α-carbon tracing of the KCNQ4 model was visualized using Chimera http://plato.cgl.ucsf.edu/chimera/[20]. Tyr270, at the N-terminus of PH, was found at the same position as the corresponding Kv1.2 residue (Ile361) (Figure 4A). We therefore assessed the electrostatic characteristics of the two models to determine if a positively charged His270 side chain might be responsible for the patient's hearing loss.

The ribbon model of wild-type KCNQ4 with the electrostatic surface potential superimposed, suggests that the side chain of Tyr270 (colored white in Figure 4B) should be electrostatically neutral. In contrast, for Tyr270His, the side chain of His270 (colored blue in Figure 4C) is predicted to retain at least a partial positive charge, which reflects a standard *p*K_a _value (6.5) for Histidine. Moreover, His270 is surrounded by the negatively charged residues Asp272, Asp266, Asp262, Glu260, and the polar residues Ser269, Ser268, and Ser265, which are capable of hydrogen bonding (all the aforementioned residues are within 10 Å of His270, Figure 4D). In hemoglobin (PDB ID: 2hhb), the side-chain *p*K_a _of His97, which is located in the loop domain between helix3 and helix4 and surrounded by negatively charged residues of Asp94 (located contiguous to the C-terminus of helix 3) and Asp99 (N-terminus of helix 4) within 5 Å distance, is shifted to an abnormally high value (pK_a _= 7.8) [21]. For these reasons, the side chain of His270 may also have a *p*K value that is greater than the standard value, and, therefore, it was predicted that more than half of the histidine side chains at position 270 carry a positive charge near physiological pH (Figure 4C).

The alteration of the electrostatic surface potential of a single helical residue in the pore region might affect the structural stability of the channel as a consequence of an alteration in the helix dipole moment. Substantial dipole moments with positive and negative unit charges at the N- and C-terminals, respectively, of α-helices are often found [21]. Comparison of three Kv channel structures suggests that the dipoles of the transmembrane helices orient the helices and are responsible, at least in part, for the structure of the pore [22-24]. The electrically neutral Tyr270, which, as noted above, is at the N-terminus of the PH, is located within 9 Å of Ala263, the S5 C-terminus (Figure 4B, C). The presence of a positively charged His adjacent to, or at the N-terminal of, an α-helix can destabilize the helix dipole [25]. Replacement of Tyr270 with His may therefore increase the dipole moment of the PH.

Alternatively, or in conjunction with the destabilization effect, His270 may impede K^+ ^transport. The electrostatic repulsion between a positively charged His270 and K^+ ^would be stronger than that between the electrically neutral Tyr270 and K^+^. In the Tyr270His model, the distance between His270 and the center pore of the channel was ~20 Å (Figure 4E, F). This distance is small enough to affect the electrostatic interaction between two charged molecules in a non-polar environment (*e.g*., the interior of a membrane protein) because the electrostatic force is strong and affects charges separated by 500-1800 Å [26-28]. Additionally, the dielectric constantsin the interior of a protein and in a lipid membrane are assumed to be 5 and 2, respectively, whereas that for bulk water is ~80 at room temperature [29]. The electrostatic potential energy for two charges in protein or lipid membrane interiors is therefore between 16 and 40 times greater than that in water. Consequently, the long-range electrostatic repulsive force between the positively charged His270 and a K^+ ^might possibly impede K^+ ^passage through the channel [30], whereas the force on K^+ ^would be smaller in the extracellular region.

Several mutations (*e.g*., Leu274His and Trp276Ser) in the KCNQ4 PH have been correlated with hearing loss [8]. The clinical features, congenital, progressive high-frequency hearing loss without substantial loss of speech recognition during the first decade of life, were the same for the patients with the aforementioned mutated KCNQ4s and the patient with Tyr270His [8,31]. Interestingly, ectopic expression of the Trp276Ser mutant and wild-type KCNQ4 in a cultured cell line caused a reduction in the channel current, whereas expression of only the mutant caused impaired trafficking of the protein to the cellular membrane [32]. The similar clinical symptoms and locations of the mutations within KCNQ4 support the proposal that Tyr270His is a pathogenic mutant associated with progressive SNHL. Further physiological and three-dimensional structural characterization of Tyr270His may identify which of our working hypotheses, structural distortion of the channel caused by a change in the PH dipole moment or electrostatic impediment of K^+ ^transport, or both, caused hearing loss in the patient, and provide insight into how to reverse hearing loss caused by KCNQ4 mutants.

## Conclusion

In summary, we identified a new KCNQ4 mutant, in which Tyr270 was replaced with His in a patient with progressive SNHL. Structural models of wild-type and Tyr270His pore regions revealed that His270 caused an alteration in the electrostatic surface potential of the PH, which, in turn, suggests that the molecular pathology of Tyr270His might cause the hearing loss in two ways. First, the increased dipole moment of the PH caused by the positively charged His270 might destabilize the PH structure, as well as that of the pore region. In parallel, the electrostatic repulsive force between His270 and K^+ ^might impede K^+ ^transport through the channel.

## Methods

### Subjects

The Research Ethics Committee of the National Tokyo Medical Center approved this study. Subjects with bilateral sensorineural hearing loss were recruited by the National Tokyo Medical Center and collaborating hospitals. Informed, written consent was obtained from the patients and their parents before removing genetic material. Medical histories and physical examinations were acquired for 127 patients whose hearing loss was not a syndromic symptom or a consequence of environmental factors (*e.g*., perinatal complication, meningitis, prenatal or postnatal drug ototoxicity, or acoustic trauma). Before sequencing the pore regions of the patients' *KCNQ4*, their mitochondrial DNA sequences (m.1555A > G or m.3243A > G) and *GJB2 *were determined to be normal (mutations in these genomic regions are known to be the most frequent causes of hereditary hearing loss [33,34]). Each patient's hearing level was examined by pure-tone audiometry. Hearing loss was classified according to pure-tone average values at 0.5, 1, 2, and 4 kHz http://hereditaryhearingloss.org/.

### Genetic analysis

Genomic DNA was extracted from blood samples using the reagents of the Gentra Puregene Blood kit (QIAGEN, Hamburg, Germany). For polymerase-chain-reaction (PCR) amplification of *KCNQ4 *exons 5, 6, and 7, the primer sets 5'-GAGATGGGGGACCTTTATCC-3' and 5'-AGCCCTACAAAGACCCTCAC-3'; 5'-GACCAGTCCTGCCTGTAACC-3' and 5'-AACTGAGCAGGAGGCAACTC-3'; and 5'-ACCCTTGCAGCCTCTTACTG-3' and 5'-CTGCTCCTAGGGCTTCTTCC-3; respectively, were used in conjunction with AmpliTaq Gold DNA polymerase (Applied Biosystems, Foster City, CA) and a PC-818 Program Temp Control System (ASTEC, Shizuoka, Japan). The PCR program was: 95°C for 4 min; 30 three-step cycles of 95°C for 30 s, 59.4°C for 30 s, and 72°C for 45 s; and then 72°C for 5 min. Primer sets for all *KCNQ4 *exons (Additional file 2: Table S1) were used to characterize the DNA encoding Tyr270His. These exons were amplified using PrimeSTAR HS DNA polymerase (Takara Bio, Shiga, Japan). All other PCR-amplification conditions were the same as mentioned above. PCR products were sequenced using an ABI 3730 DNA sequence analyzer and the reagents of the ABI Prism Big Dye Terminator Cycle Sequencing kit (Applied Biosystems). Characterization of the sequences was undertaken using SeqScape software v2.6 (Applied Biosystems) and DNASIS Pro (Hitachisoft, Tokyo, Japan), with the *KCNQ4 *sequence (NG_008139, NCBI Build37.1) as the reference. Control DNA was obtained from 96 Japanese subjects with normal hearing.

### Molecular modeling of KCNQ4

To find a voltage-gated K^+ ^channel with a sequence homologous to that of KCNQ4 that could be used as the structural template for the modeling exercise, we searched the Protein Data Bank using Gapped BLAST [13] and PDBsum (European Bioinformatics Institute: http://www.ebi.ac.uk/pdbsum/. The transmembrane helices and the voltage sensor and pore regions of KCNQ4 and Tyr270His were then modeled using SWISS-MODEL [15-17], with the crystal structure of Kv1.2 (PDB ID: 3LUT, chain B) as the template [11]. Both KCNQ4 models were built using the automatic modeling mode and default parameters. The qualities of the models were evaluated using the Verify_3D Structure Evaluation Server (see Additional file 2: Table S1) [18,19]. Finally, the models were each superimposed onto Kv1.2 using Chimera [20] to visualize the modeled α-carbon frames and ribbon models, with the electrostatic surface potentials of KCNQ4 or Tyr270His overlaid.

## Abbreviations

DFNA2: Nonsyndromic autosomal dominant sensorineural deafness type 2; KCNQ4: Potassium voltage-gated channel; KQT-like subfamily: Member 4; Kv: Voltage-gated potassium channel; PCR: Polymerase chain reaction; SNHL: Sensorineural hearing loss.

## Competing interests

The authors declare that they have no competing interests.

## Authors' contributions

KN was involved in the design of the study, performed the experiments and data analysis, and drafted the manuscript. HM was involved in the conception of the study and polished the manuscript. HK was involved in the conception of the study and gave considerations about structural information. SH provided information about the patient with sensorineural hearing loss. TM conceived the study, and participated in its coordination, design, data analysis and production of the manuscript. All authors read and approved the final manuscript.

## Supplementary Material

Additional file 1 **Figure S1**. The quality of the models was assessed by the Verify-3D program. The vertical axis indicates the average 3D-1D score for residues in 21-residue sliding window, the centre of which is at the sequence position indicated by the horizontal axis. Initial models of KCNQ4 exhibited better quality than those of the Kv1.2 template (Cys327 to Thr716). Nearly 100% of the scores were positive and most of these were higher than 0.1. Thus, we took the predicted KCNQ4 structure to be trustworthy. Click here for file

Additional file 2 **Table S1**. The primer sets for *KCNQ4*. The number of each primer set indicates the target exons of *KCNQ4*. Each primer specific for a given genomic sequence (upper case) is conjugated with either a universal forward M13 (lower case) or a reverse M13pUC (lower case). Click here for file
