# Supplementary figures and images for: In silico modeling of the pore region of a KCNQ4 missense mutant from a patient with hearing loss
Source: BMC Res Notes. 2012 Mar 15;5:145. doi: 10.1186/1756-0500-5-145 (PMC3374714; doi:10.1186/1756-0500-5-145)

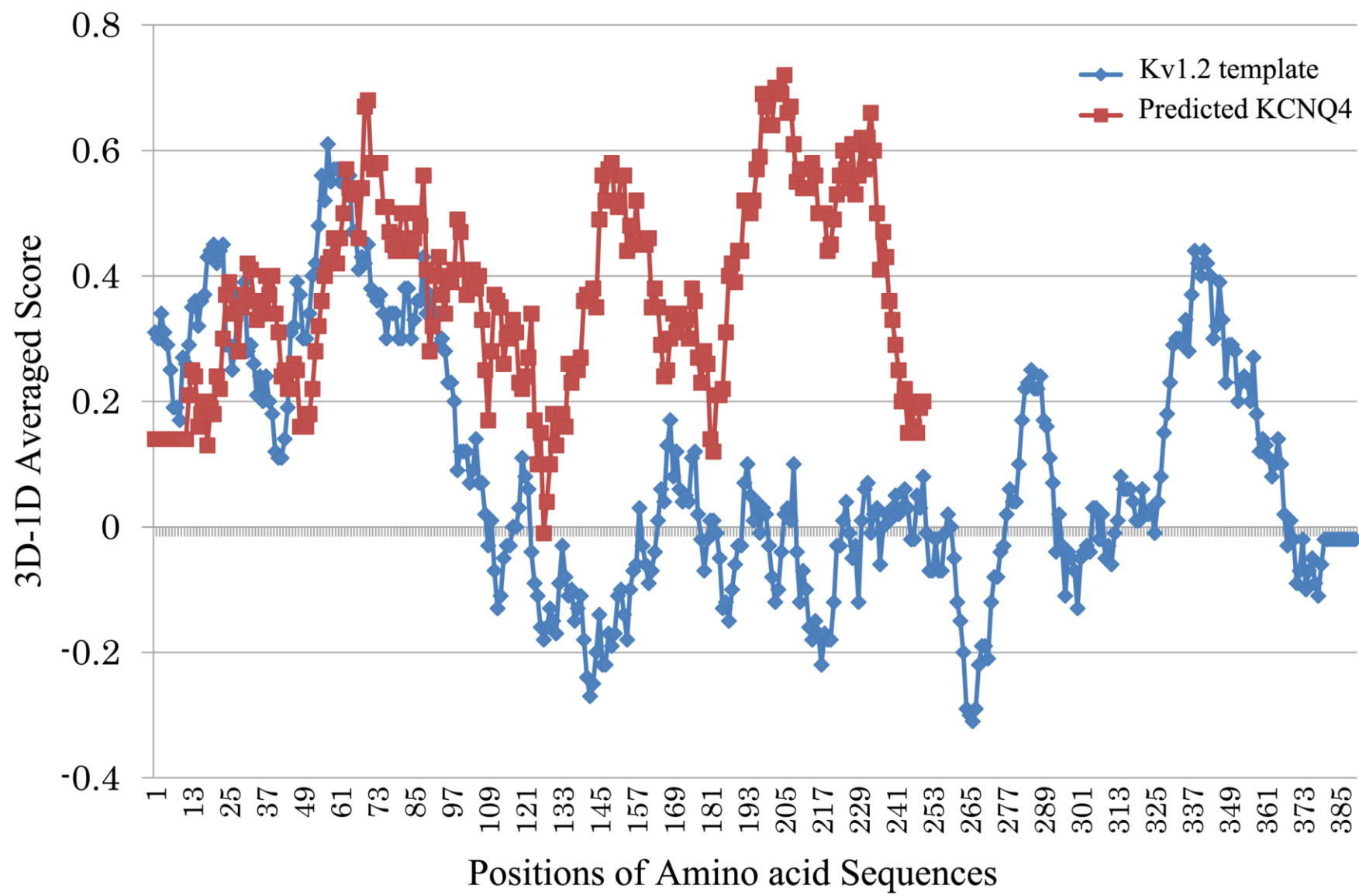

Supplement: Additional file 1 — Figure S1. The quality of the models was assessed by the Verify-3D program. The vertical axis indicates the average 3D-1D score for residues in 21-residue sliding window, the centre of which is at the sequence position indicated by the horizontal axis. Initial models of KCNQ4 exhibited better quality than those of the Kv1.2 template (Cys327 to Thr716). Nearly 100% of the scores were positive and most of these were higher than 0.1. Thus, we took the predicted KCNQ4 structure to be trustworthy. [file 1756-0500-5-145-S1.PDF]
