# Supplementary material for: In silico modeling of the pore region of a KCNQ4 missense mutant from a patient with hearing loss
Source: BMC Res Notes. 2012 Mar 15;5:145. doi: 10.1186/1756-0500-5-145 (PMC3374714; doi:10.1186/1756-0500-5-145)
Supplement: Additional file 2 — Table S1. The primer sets for KCNQ4. The number of each primer set indicates the target exons of KCNQ4. Each primer specific for a given genomic sequence (upper case) is conjugated with either a universal forward M13 (lower case) or a reverse M13pUC (lower case). [file 1756-0500-5-145-S2.PDF]

| Primer | Primer sequences (5' → 3') | PCR product (base pairs) |
|---|---|---|
| ex1-F | tgtaaaacgacggccagtAGCCATGCGTCTCTGAGC | 652 |
| ex1-R | caggaaacagctatgaccAGGTCAGAAGCGAGGTCAAGC | |
| ex2-F | tgtaaaacgacggccagtTCCACTGCCAGACCTGCTACTCA | 624 |
| ex2-R | caggaaacagctatgaccACCTTCAGCCCTCAGCCCAG | |
| ex3-F | tgtaaaacgacggccagtGCCCTCCGGAATCGTCAAGT | 635 |
| ex3-R | caggaaacagctatgacccAGGATCTGCAGGAAGCGCA | |
| ex4-F | tgtaaaacgacggccagtGAGGATGGCAGGGTCGCTTC | 623 |
| ex4-R | caggaaacagctatgaccGCCTCCTGCTAGGGCTCCAA | |
| ex8-F | tgtaaaacgacggccagtCCCTGGCTCTGACATTGATCTGC | 580 |
| ex8-R | caggaaacagctatgaccGGCAGGGCTTCACCAATGC | |
| ex9-F | tgtaaaacgacggccagtATTCTGGCCGGGCTGTCAGT | 590 |
| ex9-R | caggaaacagctatgaccGATGGGAAAGGATGGCATGGA | |
| ex10-F | tgtaaaacgacggccagtCAAGAAGTCTCCGCTTGGCG | 608 |
| ex10-R | caggaaacagctatgaccTGCCACCCTCCACGTGATTC | |
| ex11-F | tgtaaaacgacggccagtTGCAAGGTGGAACCAGTGGG | 520 |
| ex11-R | caggaaacagctatgaccAGCAGCAGTGGGTGGCACA | |
| ex12-F | tgtaaaacgacggccagtCCATTGCCCAGTCTCTGCCA | 636 |
| ex12-R | caggaaacagctatgaccCTGCAAGCCAGGGACCTCCT | |
| ex13-F | tgtaaaacgacggccagtCCTCCATCTGGCAGGGCAAT | 538 |
| ex13-R | caggaaacagctatgaccTCTGAAGGTTATGGGCGGGC | |
| ex14-F | tgtaaaacgacggccagtCCAGGCGGGATTTGTGCTTC | 602 |
| ex14-R | caggaaacagctatgaccCCTCTGTGCGACCTGGCATC | |
